# Service by Retirement

**Published:** 1912-11-16

**Authors:** 


### HOSPITAL WEAKNESS AND HOSPITAL STRENGTH.

(Contributions to this page will be welcomed.)

### II.—Service by Retirement.

It is a hard principle but a sound one that the time arrives in the life of the most zealous and successful workers for voluntary hospitals, as for all enterprises, when the greatest service such workers can render their institution is to retire from office. The hardship for the individual, if this principle is faced and reduced to its proper proportions, disappears. For when the hour for retirement arrives, to fail to retire means loss of influence. To remain on entails the risk of creating, however regretfully, a feeling that the man having done his work should give way. To suffer such sentiments to arise dims much of the glory of the good work done, and to delay still may mean its fading to the vanishing point. There are few sadder exhibitions than that of a blindness to realise the true inwardness of things. We have given some striking examples of the inestimable value of the services rendered by individuals to particular charities when describing the development and progress of important hospitals and kindred institutions. Some interesting evidence in the old days, up to thirty or possibly twenty years ago, of the effects upon hospitals of the lifelong interest and work done by individuals or members of one family has now practically disappeared. In the days we refer to it was often the case that the individual and his family would take up keenly the cause of a particular institution, work for it continuously and successfully, and so gradually acquire a paramount influence in the management and control of its affairs. These were the days of the ten-feet blackboards, on which were inscribed the names of the larger donors and benefactors. In some ways the results were wholly good, but it not infrequently happened that the supreme control exercised by one or even a few individuals with family connections diminished and prevented the interest of a large number of people being won for particular institutions. The truth is that such exclusive service or control is apt to spread abroad the impression that help of any kind from independent persons, however wealthy, might be resented.

### Possible Impediments to Progress.

Again, we could quote instances within our knowledge where an excellent man has spent the best years of his life, and continued to spend in high office many such years after he has arrived at an age when the majority of men desire to retire from active business and service. In these cases, though eminent services may have created a feeling of intense respect and even affection for the individual, it does, and, indeed, it must, happen that the ancient worker in responsible control lessens his grip, and that in this way the discipline and even the reputation of a great hospital may be imperilled. It is sad to realise, but it is true, that there is sometimes a tendency amongst ambitious stipendiaries in high position to encourage the septuagenarian to continue at the head of affairs because they are thus able to obtain uninterrupted control themselves. Where this has been the case we have found evidence of injustice, of a harsh discipline, and of other things which have combined gradually to weaken efficiency in all departments. It is well to face dangers of this kind by putting them down bluntly in black and white, for very often the ancient who causes the weakness is not conscious of the fact, so that a quiet consideration of the possibilities of this particular danger may lead to its termination. The keenest and best of workers is bound to have his own plan of work, and as years go on he is less inclined to change it from the mere habit of doing things in a certain way. This habit fosters the feeling that in fact he has become indispensable to his charity. Men and women of this type, though most excellent people within their limitations, sometimes do in fact impede the progressive development of great institutions. Further, they may diminish their financial well-being under a voluntary system like that which has made our hospitals the pride and admiration of those who know most about them.

### Sound Judgment and Personal Courage.

It is very hard and requires a great deal of courage for a man to retire from a great position in the circumstances we have just mentioned, when surrounded by those who have worked with the chief for periods of years, loving the work, and being justly proud of the results achieved. Even when the clearest-sighted worker feels the moment of retirement is approaching, the actual retirement is often prevented by the remonstrances of friends and fellow-workers. We have felt and still maintain that the true test of the value of a man's or a woman's work in the institutional world is that they can give it up and retire without such a step diminishing by a hair's breadth the efficiency, the prosperity, or the continuance of the institution or enterprise at its highest. Nobody is indispensable, and as every zealous worker appreciates sympathy and recognition, it cannot be contested that when the moment for retirement arives the wisest and the most gratifying termination to years of personal service is to withdraw from the place of honour, and, according to individual leaning, either to take a humbler place in the management or to quit it altogether.

### New Blood Needed.

New blood is essential to continuous, incessant, successful activity. Time brings many changes, more developments and increasing difficulties with the ever-recurring necessity to adopt new methods and to reorganise old systems. In such circumstances it is far better that those who are to be responsible for the efficient administration of a great charity in future years should select one of the ablest of the younger men and put him at the head of affairs. Speaking with the authority of years of experience, with the advantage of a knowledge of the views and opinion of the wealthy people who include the most generous givers to the voluntary hospitals, we can say with assurance that the reiterated and constant appeals from the same individual for gifts of money for a particular object come to be after a time less and less effective. Such appeals may even excite a feeling of resentment or create an atmosphere of irritation towards the particular object which may be so near the heart of the beggar. That is why in the old days, when the dinner was a never failing source of supplies to the hospital exchequer, a new chairman with a new connection and the recruiting of new interests was the very essence of successful appeals. Our view is that the feeling just indicated on the part of the large donors is justified and has at the bottom of it, consciously or unconsciously, a sound principle. That principle is that if the money given at intervals to a great institution by a wealthy man or woman is to provide the maximum good for the particular object, it is essential that there shall be a change from time to time in the personality of the individual who voluntarily undertakes to seek contributions and raise funds. One, and possibly the most successful of all those who have been responsible for the raising of the largest sums for hospitals in this country, has felt the force so strongly of what we have here set down in words that, in what he calls self-defence he has invariably fixed a sum of money which he would lay himself out to use his utmost endeavours to raise, and then, on the completion of this sum, he has insisted on making way for some one else to bear the burden and do the work. We hold that this view is sound for the sake of the individual worker, for it proves, as the history of his institution, if properly managed, must record, that not only did A. bring large funds to the hospital exchequer, but that he so planned his work and rendered his service that he encouraged others to enter the field. These new friends once enlisted, he has secured their increasing energies and whole influence for the charity, by putting aside all personal considerations, and retiring at the psychological moment. Men of this type invariably lift their institution higher by providing that the work shall be done, if not better at least as well as it was being done at the time of their retirement. Contrast this type of successful worker for charities with the opposite type of people who persuade themselves and insist that it is impossible for them to retire because the institution cannot go on without them. To make such a statement, much more to believe it, is in our opinion the best proof a man or woman can give that it will be well for the particular institution when their retirement is secured. We have been led to these reflections and their publication by the conviction that the present is undoubtedly a time in the history of voluntary hospitals when it will be well for every great institution that the principles here formulated shall carry due weight. Let us hope they may be weighed by all institutional workers, for so infinite benefit may result to not a few voluntary institutions.

